# Age‐associated mitochondrial complex I deficiency is linked to increased stem cell proliferation rates in the mouse colon

**DOI:** 10.1111/acel.13321

**Published:** 2021-02-24

**Authors:** Craig Stamp, Julia C. Whitehall, Anna L. M. Smith, David Houghton, Carla Bradshaw, Elizabeth A. Stoll, Alasdair P. Blain, Doug M. Turnbull, Laura C. Greaves

**Affiliations:** ^1^ Wellcome Centre for Mitochondrial Research Newcastle University Newcastle upon Tyne UK; ^2^ Biosciences Institute Newcastle University Newcastle upon Tyne UK; ^3^ Translational and Clinical Research Institute Newcastle University Newcastle upon Tyne UK

**Keywords:** aging, colon, complex I, mitochondria, stem cells

## Abstract

One of the hallmarks of aging is an accumulation of cells with defects in oxidative phosphorylation (OXPHOS) due to mutations of mitochondrial DNA (mtDNA). Rapidly dividing tissues maintained by stem cells, such as the colonic epithelium, are particularly susceptible to accumulation of OXPHOS defects over time; however, the effects on the stem cells are unknown. We have crossed a mouse model in which intestinal stem cells are labelled with EGFP (*Lgr5*‐*EGFP*‐*IRES*‐*creERT2)* with a model of accelerated mtDNA mutagenesis (*PolgA* ^mut/mut^) to investigate the effect of OXPHOS dysfunction on colonic stem cell proliferation. We show that a reduction in complex I protein levels is associated with an increased rate of stem cell cycle re‐entry. These changes in stem cell homeostasis could have significant implications for age‐associated intestinal pathogenesis.

## Graphical abstract

Age‐associated mitochondrial DNA mutations accumulate in the colonic epithelial crypts of the *PolgA* ^mut/mut^ and *PolgA* ^+/mut^ mice. This leads to a reduction in mitochondrial complex I and IV protein levels. Stem cells with complex I defects re‐enter the cell cycle 20% more frequently than those with normal complex I levels.

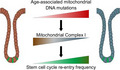

**Abbreviations**
CI: complex I
CIV: complex IV
CldU: 5‐chloro‐2′‐deoxyuridine
EGFP: enhanced green fluorescent protein
GLMM: generalized linear mixed model
IdU: 5‐iodo‐2′‐deoxyuridine
MTCO1: Cytochrome *c* oxidase subunit I
MtDNA: mitochondrial DNA
NDUFB8: NADH:ubiquinone oxidoreductase subunit B8
OXPHOS: oxidative phosphorylation
TOMM20: translocase of outer mitochondrial membrane 20
VDAC1: voltage‐dependent anion channel 1

## 1 INTRODUCTION

The accumulation of molecular damage to adult stem cells over time results in reduced capacity for self‐renewal and tissue regeneration, and is thought to play a causative role in age‐related frailty and disease. Mitochondrial dysfunction is a molecular hallmark of aging (Lopez‐Otin et al., 2013) and the generation of a progeria mouse model with an error‐prone version of the mitochondrial DNA (mtDNA) polymerase (*PolgA* ^mut/mut^ mice) provided evidence that mtDNA mutations (Kujoth et al., 2005; Trifunovic et al., 2004) and resulting defects in oxidative phosphorylation (OXPHOS) (Baines et al., 2014; Vermulst et al., 2007) could cause premature aging. Subsequent analysis of the underlying mechanisms showed that stem cell dysregulation played a major role (Ahlqvist et al., 2012; Chen et al., 2009; Fox et al., 2012). mtDNA mutations resulting in OXPHOS defects are observed in aging human stem cell populations (Fellous et al., 2009; McDonald et al., 2008; Taylor et al., 2003), including the colonic epithelium (~15% of crypts are OXPHOS‐deficient by age 70 (Greaves et al., 2010; Taylor et al., 2003)). We have recently shown that age‐associated defects in OXPHOS complexes I (CI) and IV (CIV) can increase tumour cell proliferation rates in a mouse model of intestinal tumorigenesis, accelerating colonic adenoma growth (Smith et al., 2020). However, the effects of CI and CIV deficiency on normal colonic stem cell proliferation rates are unknown.

**FIGURE 1:**
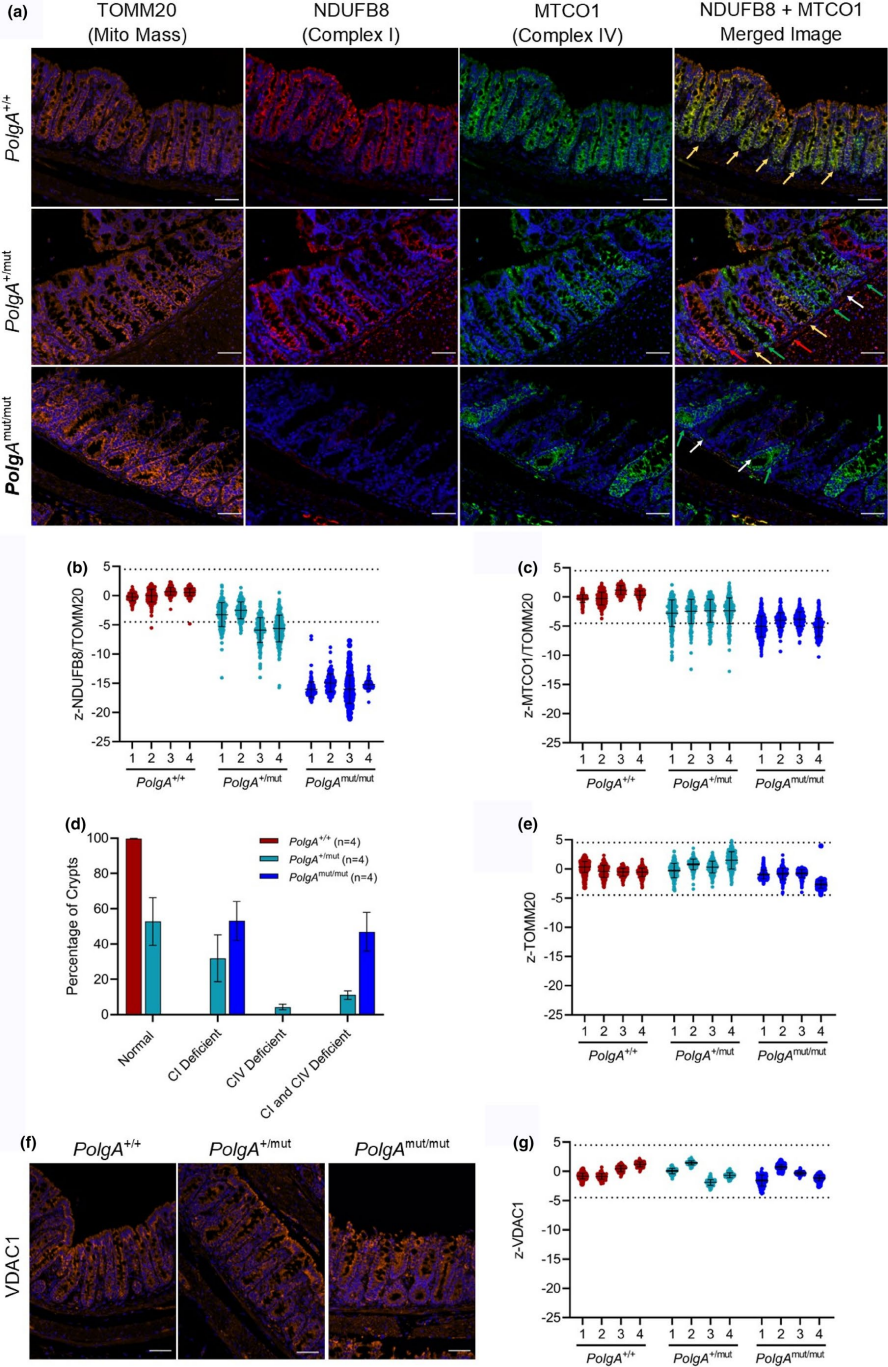
Mitochondrial OXPHOS labelling in colonic crypts of 12‐month‐old *PolgA* ^+/+^, *PolgA* ^+/mut^ and *PolgA* ^m^ ^ut/mut^ mice. (a) Immunofluorescent panel showing levels of mitochondrial NDUFB8 (CI, Alexa Fluor 647), MTCO1 (CIV, Alexa Fluor‐546) and TOMM20 (Alexa Fluor‐488). Yellow arrows: OXPHOS normal; red arrows: CIV deficiency; green arrows: CI deficiency; and white arrows: CI + CIV deficiency. Cell nuclei are labelled with DAPI (blue). Scale bars = 50 µm (b–c) Dot plots showing z‐scores of NDUFB8 and MTCO1 relative to TOMM20. Crypts with z‐score <−4.5 (dashed line) were defined as "deficient." Error bars show mean ± SD. Numbers of crypts quantified per mouse were as follows: *PolgA* ^+/+^: (l‐r) *n* = 223, 259, 306 and 332; *PolgA* ^+/mut^: (l‐r) *n* = 201, 200, 200 and 200; *PolgA* ^mut/mut^: (l‐r) *n* = 253, 191, 257 and 222. (d) The percentage of crypts per genotype with the 4 OXPHOS categories, bars show mean, and error bars are SEM. (f) Immunofluorescent images showing VDAC1 (Alexa Fluor 488) levels. Scale bars = 50 µm (g) Dot plots showing z‐scores of VDAC1 levels. *n* = 150 crypts were quantified per mouse. Error bars show mean ± SD

We investigated this by crossing the *PolgA* ^mut^^/^^mut^ mice with *Lgr5*‐*EGFP*‐*IRES*‐*creERT2* mice, which have an EGFP under the *Lgr5* (a well‐accepted stem cell marker) promoter (Barker et al., 2007). For ease, mice are referred to by their *PolgA* genotype only. Levels of NDUFB8 (CI) and MTCO1 (CIV) were quantified in *PolgA* ^+/mut^ and *Polg* ^mut/mut^ mice at 12 months of age and compared with age‐matched *PolgA* ^+/+^ controls using a validated immunofluorescent protocol (Rocha et al., 2015; Smith et al., 2020) (Figure 1a). Mean intensities were normalized to the mitochondrial mass marker TOMM20, and z‐scores relative to the *PolgA* ^+/+^ mice were generated (Figure 1b,c). Crypts with z‐scores of <−4.5 were defined as deficient. In the *PolgA* ^+/mut^ mice, 31.88% of crypts were CI‐deficient, 4.24% CIV‐deficient and 11.06% CI + CIV‐deficient. In the *PolgA* ^mut/mut^ mice, 53.1% of crypts were CI‐deficient and 46.9% CI + CIV‐deficient (Figure 1d). No crypts were classified as deficient in TOMM20 (Figure 1e), indicating no differences in mitochondrial mass. This was confirmed using a second mass marker, VDAC1 (Figure 1f,g).

**FIGURE 2:**
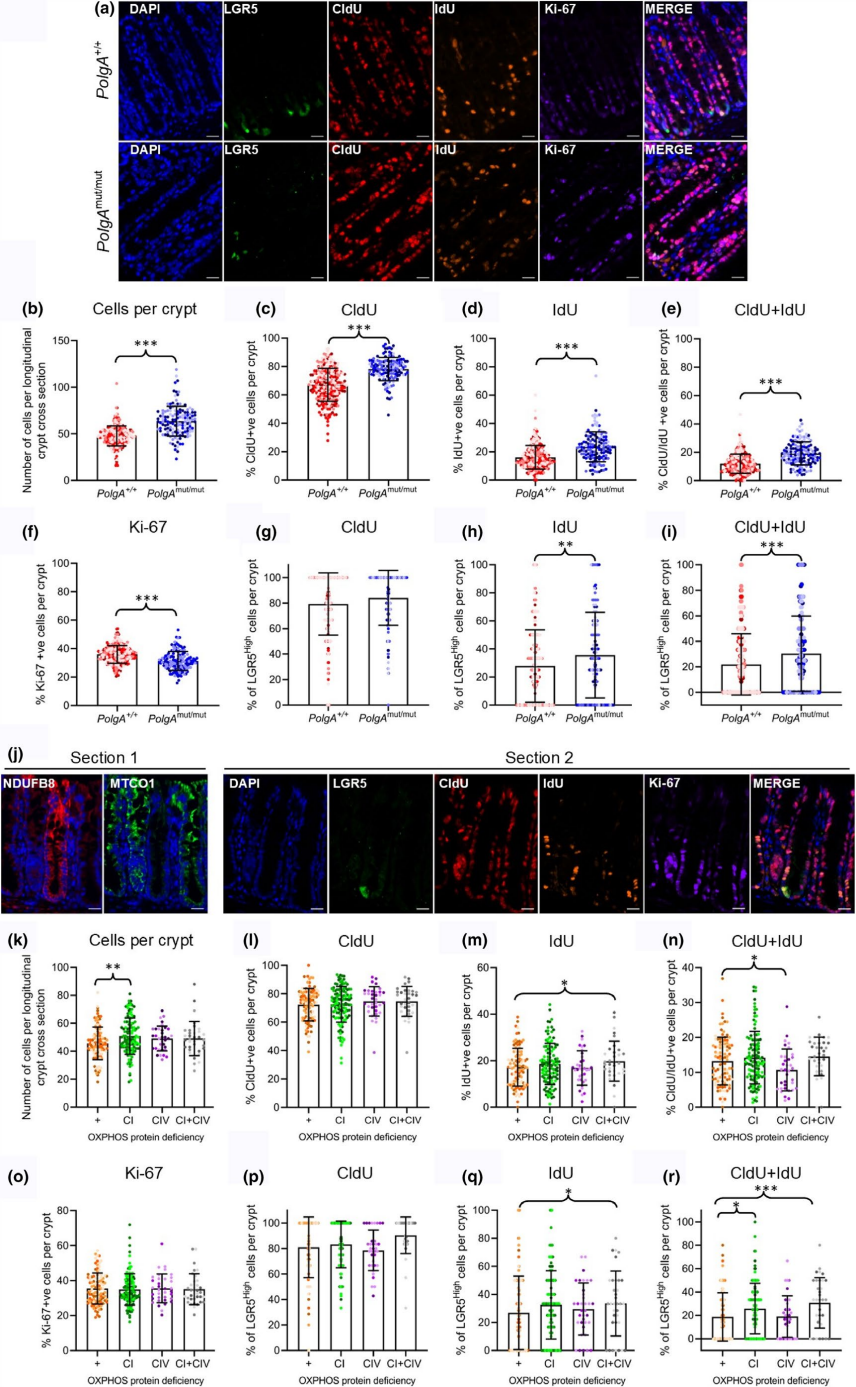
Quantification of thymidine analogue labelling in LGR5^High^ stem cells in colonic crypts of 12‐month‐old *PolgA* ^+/+^, *PolgA* ^*+/*^^*mut*^ and *PolgA* ^mut^^/^^mut^ mice. (a) Immunofluorescent panel showing LGR5 (Alexa Fluor 488), CldU (Alexa Fluor 546), IdU (Alexa Fluor 647) and Ki‐67 (Alexa Fluor 750) labelling in *PolgA* ^+/+^ and *PolgA* ^mut/mut^ mice. Scale bars = 20 µm. (b–f) Quantification of total cells, CldU, IdU, CldU + IdU and Ki‐67 labelling per crypt. (g–i) Proportion of LGR5^High^ cells per crypt incorporating CldU, IdU and CldU + IdU. (b–i) Each dot represents a single crypt and is colour‐coded by mouse ID, *n* = 4 mice per group. Numbers of crypts quantified per mouse were as follows: *PolgA* ^+/+^: *n* = 64, *n* = 81, *n* = 60 and *n* = 65; *PolgA* ^mut/mut^: *n* = 49, *n* = 24, *n* = 41 and *n* = 20. (j) Immunofluorescent panel showing MTCO1 (Alexa Fluor 546) and NDUFB8 (Alexa Fluor 647), labelling in section 1 and LGR5, CldU, IdU and Ki‐67 labelling in the adjacent section. Scale bars = 20 µm. Crypts were grouped by OXPHOS status. (k–o) Quantification of total cells, CldU, IdU, CldU + IdU and Ki‐67 labelling per crypt. (p–r) The proportion of LGR5^High^ cells per crypt incorporating CldU, IdU and CldU + IdU. (k–r) Each dot represents a single crypt and is colour‐coded by mouse ID (*n* = 4). Numbers of crypts per category: +, *n* = 100; CI, *n* = 117; CIV, *n* = 35; and CI + CIV, *n* = 34. (b–i) and (k–r) Negative binomial or Poisson GLMM, *p < 0.05, **p < 0.001. and ***p < 0.0001

Total crypt cell and LGR5^High^ stem cell proliferation indices were analysed using multiple thymidine analogue labelling (Smith et al., 2020; Stoll et al., 2011). Mice were injected daily with CldU for 4 days, and a final injection of IdU was given 15 h before death. These times were based on the reported murine colonic crypt turnover time of 4–5 days and a 24‐h stem cell cycle (Barker et al., 2007). CldU+ cells had divided at least once within the previous 4 days, IdU+ cells had divided within the previous 15 h, and those that were CldU+/IdU+ had divided at least twice within the 4‐day period (Figure 2a). All data were analysed using a Poisson (CldU, IdU, CldU + IdU and Ki‐67) or negative binomial (cells per crypt), generalized linear mixed model (GLMM) allowing variation between individual mice to be accounted for as a random effect. First, we compared cell proliferation indices between *PolgA* ^+/+^ and *PolgA* ^mut/mut^ mice. *PolgA* ^mut/mut^ mice had a significantly higher number of cells per longitudinal cross‐sectional area (*p* < 0.0001) and increased rates of CldU, IdU and CldU + IdU incorporation compared with *PolgA* ^+/+^ mice (Figure 2b–e *p* < 0.001 for all). The proportion of Ki‐67+ cells was significantly lower in *PolgA* ^mut/mut^ mice (*p* < 0.001, Figure 2f) suggesting a smaller proliferative zone with a higher rate of cell turnover. Specifically comparing LGR5^High^ stem cells per crypt from the *PolgA* ^+/+^ and *PolgA* ^mut/mut^ mice, there was no significant difference in the proportion of CldU incorporation; however, a significantly higher proportion of *PolgA^mut^* ^/^ *^mut^* LGR5^High^ stem cells incorporated IdU (*p* = 0.0189) and CldU+IdU compared with *PolgA* ^+/+^ mice (*p* < 0.001, Figure 2g–i). Next, we focussed on the *PolgA* ^+/^ *^mut^* mice, exploiting the mosaic pattern of OXPHOS defects to make intra‐mouse comparisons. Three serial sections were analysed: CI and CIV levels were quantified in the first; thymidine analogue incorporation and Ki‐67 labelling were quantified in the second (Figure 2j); and the OXPHOS status was confirmed in the third. Crypts in the "OXPHOS normal" category were used as controls, and all comparisons were made to this group. There were significantly more cells per longitudinal crypt section in CI‐deficient crypts (*p* = 0.0017) compared with OXPHOS normal crypts, but no changes in CIV‐ or CI + CIV‐deficient crypts (Figure 2k). There were no significant differences in the CldU incorporation between the groups (Figure 2l), but there was a higher proportion of cells with IdU incorporation in crypts with CI + IV deficiency (*p* = 0.033, Figure 2m). Interestingly, there was a significantly lower proportion of CldU + IdU incorporation in cells in CIV‐deficient crypts (*p* = 0.0317, Figure 2n) suggesting that isolated CIV deficiency increases the time between successive cell cycles. Ki‐67 labelling was equivalent between all groups (Figure 2o). Next, we focussed on the LGR5^High^ cells. There were no significant changes in CldU incorporation in LGR5^High^ stem cells in the presence of any of the OXPHOS defects (Figure 2p); however, LGR5^High^ cells with combined CI and IV deficiency had significantly higher IdU incorporation compared with OXPHOS normal cells (*p* = 0.0386, Figure 2q). There was a significantly higher frequency of CldU + IdU incorporation in LGR5^High^ stem cells with either isolated CI deficiency (*p* = 0.0373) or combined CI + CIV deficiency (*p* = 0.00069) compared with those with normal OXPHOS protein levels (Figure 2r). These data suggest that CI deficiency is driving a ~20% increase in LGR5^High^ stem cell division rates in the colon.

We have directly examined the effect of age‐related OXPHOS defects on colonic stem cell proliferation rates *in vivo* and show for the first time that CI deficiency results in an increased frequency of cell cycle re‐entry in LGR5^High^ stem cells of the colon of *PolgA* ^+/mut^ and *PolgA* ^mut/mut^ mice. These data could either reflect a general increase in stem cell division rate or a change in the ratio of rapidly dividing stem cells to non‐dividing or slowly dividing stem cells within the stem cell niche (Barriga et al., 2017). Despite certain limitations, such as the model‐specific mechanism of mutation and mutational spectrum of the *PolgA* mice (Kujoth et al., 2005; Trifunovic et al., 2004), the functional consequences of the increased rate of mutagenesis (CI and CIV deficiency) make them a useful model in which to study the effects of defects in these proteins in stem cells. Previous work looking at cell cycle progression in the small intestine of *PolgA* ^mut/mut^ mice at 3 months of age showed a decrease in proliferation rates, suggesting there are either differential effects of OXPHOS deficiency between the colon and small intestine, or adaptive changes with age. Our previous studies have shown an increasing upregulation of *d* *e novo* serine synthesis and one‐carbon metabolic pathway enzymes in the *PolgA* ^mut/mut^ colon from 6 months onwards (Smith et al., 2020). These pathways are associated with biomass production in rapidly dividing cells and may influence stem cell division rates (Maddocks et al., 2017). We do not know the molecular effects of CI deficiency on LGR5^High^ stem cell function beyond the changes in proliferation rates shown by this study; however, given the importance of control of the stem cell compartment to maintain epithelial crypt homeostasis, further work is required to determine the underlying molecular mechanisms.

## 2 EXPERIMENTAL PROCEDURES

### 2.1 Animals

Female mice were group‐housed in individually ventilated cages at 25°C with a 12‐h light/dark cycle. All mice were on a C57BL6/J background. Animal experiments were conducted in compliance with the UK Home Office (PPL P3052AD70) and the Newcastle University Animal Welfare Ethical Review Board (AWERB 425).

### 2.2 Thymidine analogue labelling

Mice (*n* = 4 per group, age: 49–51 weeks) were injected with 50 mg/kg of CldU at 10am and 6 pm on days 1–3. On day 4, they were injected with 50 mg/kg of CldU at 10am followed by 50 mg/kg IdU at 6 pm (Smith et al., 2020). Mice were humanely killed by cervical dislocation 15 h later. The intestines were removed and fixed in 10% neutral buffered formalin for 24 h before standard dehydration and paraffin embedding.

### 2.3 Immunofluorescence

OXPHOS immunofluorescence was performed on 3‐µm serial sections of colon using the same antibodies as previously described (Rocha et al., 2015), and immunofluorescence was performed to detect the thymidine analogues as described (Smith et al., 2020). Antibody specificity was determined using mice singly injected with either CldU or IdU, and no cross‐reactivity was observed. Sections were imaged using a Zeiss Axio Imager M1 fluorescent microscope and labelled proteins quantified as previously described (Rocha et al., 2015). For the thymidine analogue labelling analysis, crypts were selected where a full longitudinal section was visible from crypt base to apex. The total number of cells per crypt, and the number of LGR5^High^, Ki‐67+, CldU+ and IdU+ cells per crypt were counted. GLMMs were performed using R programming, code is available on request.

## CONFLICT OF INTEREST

The authors declare no competing financial interests.

## AUTHOR CONTRIBUTIONS

CS and CB bred and maintained the mice. CS, JCW, DH ALMS and EAS performed experimental work and collected data. JCW and APB performed statistical analysis. DMT and LCG designed the study, supervised the project and wrote the manuscript.

## Data Availability

Source data for this study are available from the corresponding author upon request.
